# Metabolomic biomarkers of pancreatic cancer: a meta-analysis study

**DOI:** 10.18632/oncotarget.20324

**Published:** 2017-08-18

**Authors:** Khyati Y. Mehta, Hung-Jen Wu, Smrithi S. Menon, Yassi Fallah, Xiaogang Zhong, Nasser Rizk, Keith Unger, Mark Mapstone, Massimo S. Fiandaca, Howard J. Federoff, Amrita K. Cheema

**Affiliations:** ^1^ Department of Oncology, Georgetown University Medical Center, Washington, DC, United States of America; ^2^ Department of Biostatistics Bioinformatics and Biomathematics, Georgetown University, Washington, DC, United States of America; ^3^ Department of Health Sciences, Qatar University, Doha, Qatar; ^4^ Lombardi Comprehensive Cancer Center, Med-Star Georgetown University Hospital, Washington, DC, United States of America; ^5^ Department of Neurology, University of California, Irvine, CA, United States of America; ^6^ Department of Neurological Surgery, University of California, Irvine, CA, United States of America; ^7^ Department of Biochemistry and Molecular and Cellular Biology, Georgetown University Medical Center, Washington, DC, United States of America

**Keywords:** pancreatic cancer, biomarkers, metabolomics

## Abstract

Pancreatic cancer (PC) is an aggressive disease with high mortality rates, however, there is no blood test for early detection and diagnosis of this disease. Several research groups have reported on metabolomics based clinical investigations to identify biomarkers of PC, however there is a lack of a centralized metabolite biomarker repository that can be used for meta-analysis and biomarker validation. Furthermore, since the incidence of PC is associated with metabolic syndrome and Type 2 diabetes mellitus (T2DM), there is a need to uncouple these common metabolic dysregulations that may otherwise diminish the clinical utility of metabolomic biosignatures. Here, we attempted to externally replicate proposed metabolite biomarkers of PC reported by several other groups in an independent group of PC subjects. Our study design included a T2DM cohort that was used as a non-cancer control and a separate cohort diagnosed with colorectal cancer (CRC), as a cancer disease control to eliminate possible generic biomarkers of cancer. We used targeted mass spectrometry for quantitation of literature-curated metabolite markers and identified a biomarker panel that discriminates between normal controls (NC) and PC patients with high accuracy. Further evaluation of our model with CRC, however, showed a drop in specificity for the PC biomarker panel. Taken together, our study underscores the need for a more robust study design for cancer biomarker studies so as to maximize the translational value and clinical implementation.

## INTRODUCTION

In 2017 an estimated 53,670 people in the US will be diagnosed with pancreatic cancer (PC), and 43,090 people will die from it [1]. These numbers underscore high disease-associated mortality. PC represents 90% of pancreatic neoplasms and is likely to become the second most deadly cancer by 2020 [2–4]. The majority of patients present with incurable disease, and the median survival for advanced or metastatic PC is less than 5% at 5 years [5]. While known risk factors include age, chronic pancreatitis, and Type 2 diabetes mellitus (T2DM), the disease, for the most part, progresses indolently. Additionally, several epidemiological variables, such as tobacco smoking and obesity, have been deemed to increase risk, however these are not specific to PC and hence cannot be used to define a high risk population for PC screening [6–8]. The only approved serum PC biomarker, CA19-9, is nonspecific and only elevated in advanced disease and hence has no role as a standalone marker [9], or for detection of early disease. Thus, there is a critical need to develop diagnostic and prognostic biomarkers with potential clinical utility. Despite extensive efforts towards development of biomarkers for PC [10–31], there is a paucity of a validated biomarker panel that is specific for PC [32–34].

Metabolomics methodology aims to identify and estimate the relative changes in the abundance of endogenous metabolites in health and disease, thus supporting the identification of biomarkers and potential targets for the development of new therapeutics [35–37]. Given the role of the pancreas as a major metabolic organ, it is reasonable to assume that the identification, characterization and validation of novel disease stratification criteria based on metabolic profiles offers a strategic advantage for PC research [38–47]. Although a number of independent groups have reported on blood based metabolomics biomarkers of PC, the lack of cross-validation limits the clinical utility of these putative biosignatures.

This study was designed to address two existing challenges in the field of biomarker research for PC. Firstly, a majority of PC patients report glucose intolerance or T2DM [48, 49]. The majority of the published PC biomarker studies, however, have not used a diabetic cohort to identify shared pathway dysregulations that would help obviate non-specific biomarkers upfront. Secondly, certain oncogenic processes, like inflammation, cachexia and oxidative stress, accompany most malignancies. However, most cancer biomarker studies, rely on a case-control study design that does not account for these confounders [50]. Thus, in the absence of cancer disease control cohorts, it is difficult to select biomarkers that are specific for a given cancer type, much less in advance of disease diagnosis or in defining risk of developing the specific neoplasm. As such, determination of predictive value of a biomarker panel without ascertaining disease specificity and cross-validation, is likely to impact subsequent clinical implementation.

Herein, we report a novel experimental evaluation of putative metabolite biomarkers for PC that were curated from an exhaustive literature survey. We used targeted mass spectrometry for relative quantification of common blood based metabolite markers of PC, found to overlap in independently conducted studies. Next, we evaluated the performance of this group of metabolites in control cohorts, including a colorectal cancer cohort (CRC) that was used as a cancer control cohort, and T2DM which was used as a non-cancer disease control cohort. Although, the constructed 10 metabolite panel provided a high accuracy classifier for delineating PC patients from NC (AUC = 0.99), the AUCs still remained high when NCs were compared to the CRC cohort, thus diminishing the specificity of this panel for PC. Interestingly, the overlap of dysregulated metabolites between the PC and T2DM cohorts was lower than that observed for PC vs CRC cohorts. Taken together, our results emphasize the need for a more rigorous prospective study design that may help eliminate non-specific metabolite markers upfront, thereby augmenting the development of classifiers with high specificity for a particular malignancy. The approach described here is broadly applicable for cancer biomarker studies.

## RESULTS

### Literature mining for delineating biomarkers of PC

Since there is no central biomarker repository that can be accessed for metabolite biomarker evaluation of PC, our first goal was to perform an exhaustive literature search to create a compendium of metabolite biomarkers of PC [14]. While there are a large number of reported studies for PC biomarkers (a keyword search for “pancreatic cancer and biomarkers” in PUBMED returns > 9500 published articles); narrowing the search with specific keywords such as “metabolomics and pancreatic cancer biomarkers”, returned approximately 44 hits on PUBMED. We also looked in data repositories such as EDRN and GDOC for a compendium of PC biomarkers, but we did not find any datasets, emphasizing that the application of metabolomics for PC biomarker research is relatively new. Furthermore, bulk of PC biomarkers studies using a metabolomics approach were published in years 2010 to 2016, underscoring the emerging nature of this field of research.

Following literature search, biomarkers were stratified based on the type of matrix used for biomarker discovery (ex. tissue, saliva, cell lines, blood or urine). We focused on blood based metabolite markers for meta-analysis (Table 1 and Supplementary Table 1), while all non-blood based studies, such as tissue, urine, and saliva, were consolidated separately (Supplementary Table 2). All blood based metabolite markers that overlapped in two or more studies were deemed significant for further evaluation and were compiled along with the comparative groups and direction of regulation as reported (Table 1). Approximately 56 metabolite markers that were found to be significantly dysregulated in blood samples obtained from “at-diagnosis” PC patients as compared to normal controls, uniquely reported by one research group, are listed in Supplementary Table 1. In addition, studies that used high risk cohorts (such as benign pancreatic conditions and chronic pancreatitis) as a reference group for delineating PC biomarkers are detailed in Supplementary Table 1 showing some overlap with those listed in Table 1.

**Table 1:** Compendium of blood based metabolite markers that overlapped between independently conducted case- control biomarkers studies of PC

Biomarker	Comparison Groups	Instrument	Matrix	Reference
1,5-anhydro-d-glucitol↓^b^	PC (*n* = 200) vs NC (*n* =200)	LC–TOFMS, GC–TOFMS	plasma	[82]
	PC (*n* = 43) vs NC (*n* =42)	GC/MS	serum	[65]
3-hydroxybutyrate↓	PC (*n* = 17) vs NC (*n* =23)	1H NMR	Serum	[38]
	PC (*n* = 19) vs NC (*n* =20)^c^	H-NMR	Blood/plasma	[64]
Alanine ↓	PC (*n* = 19) vs NC (*n* =20)^c^	H-NMR	Blood/plasma	[64]
	PC (*n* = 360) vs NC (*n* =8372)^c^	HPLC-ESI-MS	plasma	[63]
Asparagine^a^	PC (*n* = 43) vs NC (*n* =42)	GC/MS	Serum	[65]
	PC (*n* = 20) vs NC (*n* =9)	GC/MS	Serum	[66]
	PC (*n* = 360) vs NC (*n* =8372)^c^	HPLC-ESI-MS	plasma	[63]
CA19-9 ↑ ^b^	PC (*n* = 84) vs NC (*n* =99)	tandem mass spectrometry	Serum	[83]
	PC (*n* = 50) vs NC (*n* =50)^c^	ELISA	serum	[84]
Choline^a^	PC (*n* = 14) vs NC (*n* =14)	1H NMR, TOCSY, HMQC or HSQC	serum	[85]
	PC (*n* = 200) vs NC (*n* =200)	LC–TOFMS and GC–TOFMS	plasma	[82]
Glutamate^a^	PC (*n* = 19) vs NC (*n* =20)^c^	H-NMR	Blood/plasma	[64]
	PC (*n* = 200) vs NC (*n* =200)	LC–TOFMS and GC–TOFMS	plasma	[82]
Glutamine ↓	PC (*n* = 5) vs NC (*n* = 2)^c^	HILIC-LC/MS RP-LC/MS	plasma	[86]
	PC (*n* = 19) vs NC (*n* =20)^c^	H-NMR	Blood/plasma	[64]
	PC (*n* = 43) vs NC (*n* =42)	GC/MS	Serum	[65]
Histidine ↓	PC (*n* = 19) vs NC (*n* =20)^c^	H-NMR	Blood/plasma	[64]
	PC (*n* = 43) vs NC (*n* =42)	GC/MS	Serum	[65]
	PC (*n* = 360) vs NC (*n* =8372)^c^	HPLC-ESI-MS	plasma	[63]
Isoleucine ↑	PC (*n* = 17) vs NC (*n* =23)	1H NMR	Serum	[38]
	PC (*n* = 19) vs NC (*n* =20)^c^	H-NMR	Blood/plasma	[64]
Lactate^a^	PC (*n* = 17) vs NC (*n* =23)	1H NMR	Serum	[38]
	PC (*n* = 19) vs NC (*n* =20)^c^	H-NMR	Blood/plasma	[64]
	PC (*n* = 20) vs NC (*n* =9)	GC/MS	serum	[66]
Leucine^a^	PC (*n* = 17) vs NC (*n* =23)	1H NMR	Serum	[38]
	PC (*n* = 360) vs NC (*n* =8372)^c^	HPLC-ESI-MS	plasma	[63]
Lysine^a^	PC (*n* = 5) vs NC (*n* = 2)^c^	HILIC-LC/MS	plasma	[86]
	PC (*n* = 19) vs NC (*n* =20)^c^	H-NMR	Blood/plasma	[64]
	PC (*n* = 43) vs NC (*n* =42)	GC/MS	Serum	[65]
	PC (*n* = 360) vs NC (*n* =8372)^c^	HPLC-ESI-MS	plasma	[63]
LysoPC(18:2)^a^	PC (*n* = 5) vs NC (*n* = 2)^c^	RP-LC/MS	plasma	[86]
	PC (*n* = 40) vs NC (*n* =50)	FI-FTICR-MS	serum	[87]
Methionine ↓	PC (*n* = 360) vs NC (*n* =8372)^c^	HPLC-ESI-MS	plasma	[63]
	PC (*n* = 43) vs NC (*n* =42)	GC/MS	Serum	[65]
Phenylalanine^a^	PC (*n* = 360) vs NC (*n* =8372)^c^	HPLC-ESI-MS	plasma	[63]
	PC (*n* = 5) vs NC (*n* = 2)^c^	RP-LC/MS	plasma	[86]
Palmitic acid ↓	PC (*n* = 40) vs NC (*n* =40)	LC-MS/MS	serum	[57]
	PC (*n* = 20) vs NC (*n* =9)	GC/MS	serum	[66]
PC-594 ↓^b^	PC (*n* = 84) vs NC (*n* =99)	tandem mass spectrometry	Serum	[83]
	PC (*n* = 40) vs NC (*n* =50)	FI-FTICR-MS	serum	[87]
Threonine ↓	PC (*n* = 360) vs NC (*n* =8372)^c^	HPLC-ESI-MS	plasma	[63]
	PC (*n* = 43) vs NC (*n* =42)	GC/MS	Serum	[65]
Tyrosine ↓	PC (*n* = 360) vs NC (*n* =8372)^c^	HPLC-ESI-MS	plasma	[63]
	PC (*n* = 43) vs NC (*n* =42)	GC/MS	Serum	[65]
Valine ↓	PC (*n* = 19) vs NC (*n* =20)^c^	H-NMR	Blood/plasma	[64]
	PC (*n* = 43) vs NC (*n* =42)	GC/MS	Serum	[65]
	PC (*n* = 360) vs NC (*n* =8372)^c^	HPLC-ESI-MS	plasma	[63]

^a^Metabolites that were found in multiple studies but were non-concordant.

^b^Metabolites that were found in multiple studies but were not included in our analysis.

^c^PC vs Chronic pancreatitis.

### Experimental evaluation of blood based biomarkers of PC

We chose blood-based metabolites for meta-analysis since it is a non-invasive matrix enabling easy sample collection. Furthermore it is amenable to the development of an assay that can be translated into clinical use with relative ease and in a cost-effective manner as compared to a more invasive procedure like tissue biopsy.

**Figure 1:**
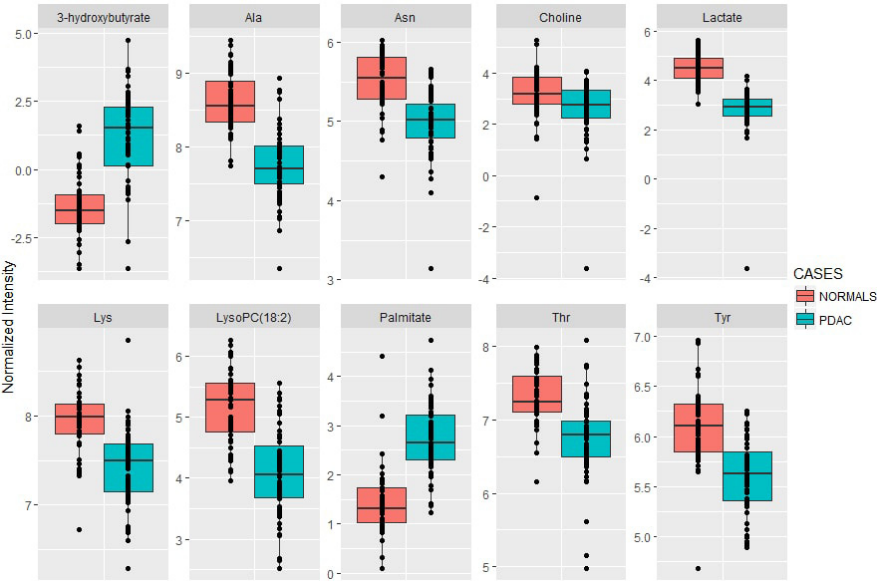
Boxplots for the ten metabolite panel Group separation based on normalized abundance of the ten dysregulated metabolites in PC as compared to NC. Solid line represents median value.

Our study included plasma samples from four diagnostic groups including PC (*N* = 59), normal controls (*N* = 48), CRC (*N* = 66) and T2DM (*N* = 19) that were used for performance evaluation of literature curated biomarkers. PC and CRC groups had localized cancer at the time of sample collection and were not previously treated with cancer therapies. The underlying rationale for choosing the latter two cohorts (CRC and T2DM), was to evaluate biomarker specificity for PC. We used stable isotope labeling and multiple reaction monitoring (SID-MRM) based targeted mass spectrometry (MS), for measuring relative abundance for 18 metabolites (of the reported 21 listed in Table 1). Three metabolites, namely 1,5-anhydro-d-gluticol, CA19-9, and PC-594 were not quantified in our panel, and hence were not used in subsequent analyses. Test of significance between the control and PC groups showed that, seventeen metabolites had a significant *p*-value after applying multiple testing correction. Next we computed relative ratios between the normal controls and PC to select ten metabolites that showed a relative fold change of greater than 1.8 or less than 0.7. This is an arbitrary cut-off and was used primarily because it has been used in several “omics” based biomarker studies [51]. Metabolite marker selection based on *p*-value and fold change helped delineate a ten metabolite panel (Table 2). Our analysis showed that eight of the ten metabolites were down-regulated in PC, including lactate, LysoPC (18:2), alanine, choline, threonine, asparagine, tyrosine, and lysine. Palmitate and 3-hydroxybutyrate were upregulated in PC when compared to normal controls. Group separation between PC and normal controls for each of the significant metabolites was visualized as box plots that were generated using in-house R scripts (Figure 1).

**Table 2:** Experimental validation of a ten metabolite PC biomarker panel curated from literature

	NC vs PC		NC vs CRC		NC vs T2DM		T2DM vs PC		CRC vs PC	
Metabolite	FDR	Fold Change	FDR	Fold Change	FDR	Fold Change	FDR	Fold Change	FDR	Fold Change
*Lactate	8.66e-15	0.33(↓)	5.25e-13	0.39(↓)	1.48e-07	2.19(↑)	1.22e-15	0.15 (↓)	0.52	0.84
*LysoPC(18:2)	1.45e-12	0.49(↓)	7.33e-16	0.46(↓)	0.55	1.05	6.80e-9	0.47 (↓)	0.94	1.06
Alanine	2.73e-16	0.56(↓)	3.67e-20	0.54(↓)	0.14	1.12	1.13e-12	0.49 (↓)	0.94	1.03
*Choline	0.01	0.68(↓)	0.0003	0.58(↓)	0.21	1.14	0.0019	0.59 (↓)	0.52	1.16
Threonine	1.15e-08	0.69 (↓)	6.40e-09	0.72	0.14	1.11	1.44e-7	0.62 (↓)	0.52	0.95
*Asparagine	7.58e-10	0.70(↓)	3.70e-06	0.79	2.59e-17	0.32(↓)	1.14e-12	2.16 (↑)	0.059	0.88
Tyrosine	1.07e-09	0.70(↓)	3.04e-13	0.66(↓)	0.81	1.01	2.38e-7	0.69 (↓)	0.52	1.05
*Lysine	1.49e-09	0.70(↓)	1.43e-10	0.70(↓)	0.04	1.15	1.032e-9	0.61 (↓)	0.94	1.00
Palmitate	2.47e-15	2.35(↑)	6.09e-12	3.60(↑)	0.007	2.47 (↑)	0.019	0.95	0.94	0.65(↓)
3-hydroxybutyrate	4.64e-13	6.91(↑)	9.29e-23	17.19(↑)	0.88	1.20	2.49e-7	5.77 (↑)	4.59e-4	0.40(↓)

Metabolites marked with an asterix are not concordant across reported studies.

**Figure 2:**
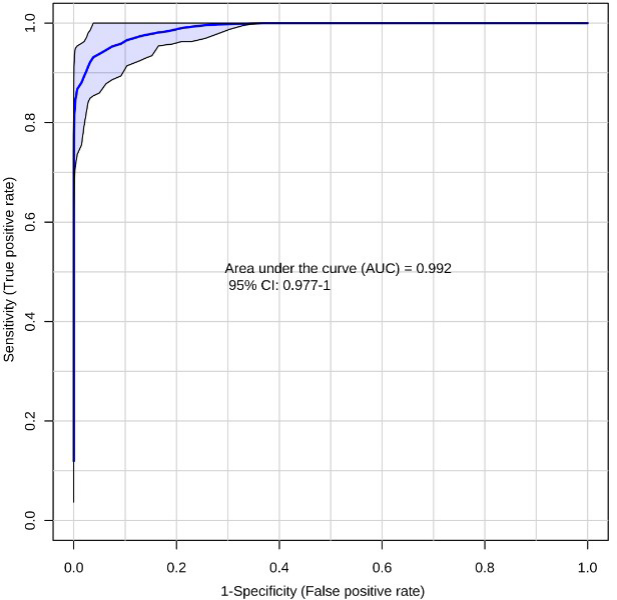
Receiver-operating characteristic (ROC) curve for PC (*n* = 59) vs NC (*n* = 48) using the ten metabolite panel yields AUC = 0.992

**Figure 3:**
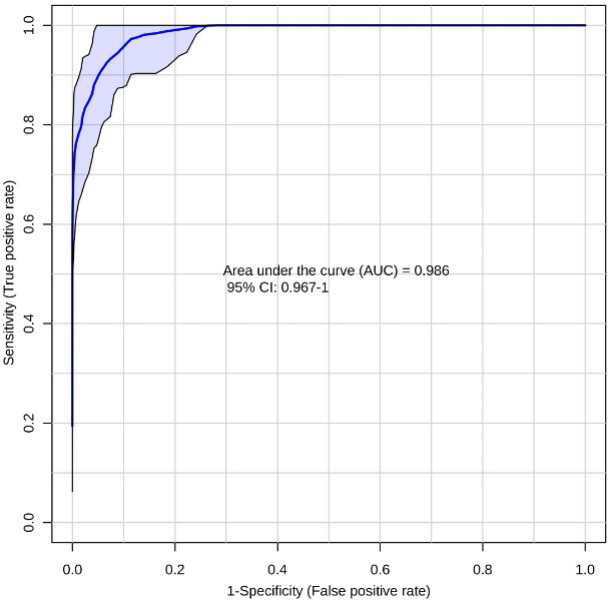
Receiver-operating characteristic (ROC) curve for CRC (*n* = 66) vs NC (*n* = 48) using the ten metabolite panel yields AUC = 0.986

**Figure 4:**
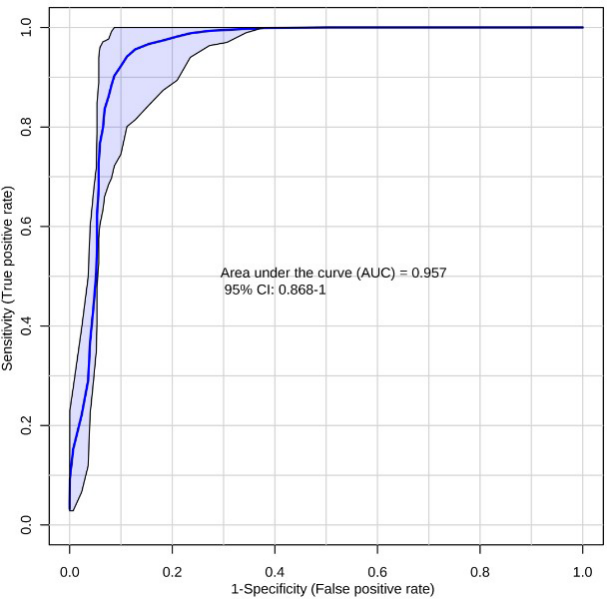
Receiver-operating characteristic (ROC) curve for T2DM (*n* = 19) vs NC (*n* = 48) using the ten metabolite panel yields AUC = 0.957

**Figure 5:**
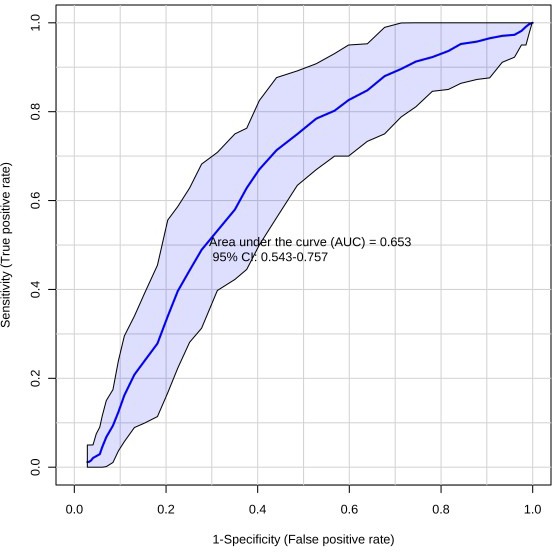
Receiver-operating characteristic (ROC) curve for CRC (*n* = 66) vs PC (*n* = 59) using the ten metabolite panel yields AUC = 0.653

Next we evaluated the efficiency of the ten-biomarker panel using biomarker analysis module of MetaboAnalyst v3.0 [52]. A random forest based multivariate ROC analysis using t-statistics for metabolite ranking resulted in a panel yielding an AUC of 0.992 (Figure 2) for PC, emphasizing high accuracy of this literature-curated classifier. Subsequently we used control cohorts to test biomarker panel specificity. Test of significance for all comparisons are detailed in Table 2. ROC analysis using the same parameters for the ten metabolites yielded an AUC of 0.986 (Figure 3) for colorectal cancer vs NC, and an AUC of 0.957 (Figure 4) for T2DM vs NC. Strikingly, test of significance for CRC vs NC (based on *p*-value and fold change cut-offs), yielded 8 of the 10 metabolites as being significant and the direction of regulation was also concordant as in NC vs PC (asparagine and threonine were non-significant). These results suggest a high degree of correlation between the two cancer malignancies with respect to metabolic dysregulation (> 75%) evidenced through our metabolite panel. Not surprisingly, the ten-metabolite panel proved to be a poor classifier for CRC vs PC (AUC = 0.65) (Figure 5).

**Figure 6:**
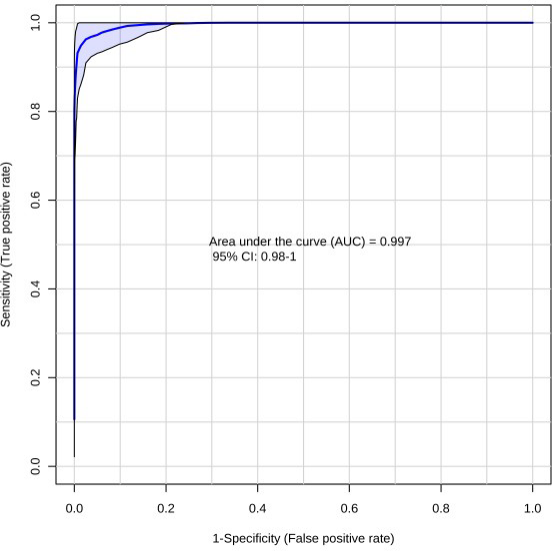
Receiver-operating characteristic (ROC) curve T2DM (*n* = 19) vs PC (*n* = 59) using the ten metabolite panel yields AUC = 0.997

We also compared T2DM cohort (*N* = 19) against normal controls, finding 3 metabolites (asparagine, lactate and palmitate) of the 10 to be significantly dysregulated between these two groups. The ROC curve generated using the ten-metabolite panel discriminated T2DM patients from PC patients with high accuracy (AUC = 0.997) (Figure 6). In order to assess if age or gender can significantly improve prediction of patient groups, the receiver operating characteristic (ROC) curves of the ten metabolite panel with age or gender are compared with the ROC curves of the panel only using three different statistical methods namely Delong, Bootstrap and Venkatraman tests [53–55]. *P*-values less than 0.05 are considered statistically significant. Our results show that all the three tests have consistent results for the ROC curves comparisons, both age and sex did not help change the ROC curves significantly for NC vs PDAC, NC vs CRC and NC vs T2DM, only age helped to change the ROC curve and improve the AUC value significantly for PDAC vs CRC.

**Figure 7:**
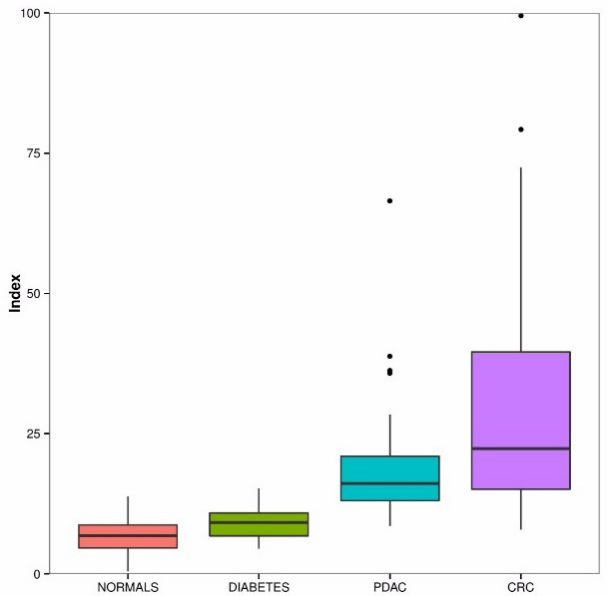
Boxplot depiction of the plasma 10 metabolite index (P10MI) Solid black horizontal line within the boxplots represents mean value.

A multinomial logistic regression model with the 10 blood (serum/plasma) analytes and patient's age was used to generate a plasma 10 metabolite index (P10MI) in order to distinguish between PC, CRC, T2DM and normal control groups (Figure 7). Higher calculated index values are associated with a higher risk of PC diagnosis, with confidence transitioning from 90% to 100% at 12.5. Based on calculated P10MI in our dataset, a value ≥ 12.5 represents a true PC case, with a 100% risk of being diagnosed with PC. Notably, there was relatively low variability of the P10MI for both the NC and the T2DM groups, with low overlap, with the PC group (dots outside error bars). On the other hand, there was a significant overlap between the PC and CRC groups, suggesting a lack of specificity of this panel for PC alone.

## DISCUSSION

The PRoBE design for biomarker development described by Pepe et al. involves biomarker discovery, followed by rigorous evaluation of biomarker performance, and finally its impact on predicting clinical outcomes [56]. Despite an array of biomarker studies of PC, there is a paucity of predictive or prognostic biomarker panels of PC that have received regulatory approvals. Moreover, PC has a relatively low prevalence (10/100,000 individuals in the USA), hence it is imperative that a biomarker panel of PC has a high specificity, thereby limiting false positives when screening a high risk population. Although there are a number of metabolomics-based PC biomarker studies, most have used (“at-diagnosis”) case-control designs that are subject to serious selection bias, limiting their general applicability to high risk populations [57, 58]. Developing a specific and sensitive panel of biomarkers offers a pragmatic approach towards increasing overall survival rates while identifying putative molecular targets for therapeutic development, and improving treatment strategies and clinical outcomes. Delineation of clinically useful biomarkers, however, requires implementation of well-designed studies for biomarker discovery followed by rigorous and blinded external validation for evaluating classification accuracy [59–62].

Creating a compendium of existing biomarker data and performing meta-analyses represent the first steps in the development of clinical assays for PC diagnosis and prognosis. Herein, we performed an exhaustive literature search to find PC case-control studies reporting dysregulated metabolites associated with PC, primarily from three different sources: PubMed, EDRN and GDOC (Georgetown Database of Cancer), using different search keywords. We delineated 21 metabolites (that included amino acids, glycerophospholipids, fatty acids and small organic acids) that were reported by two or more research groups as being dysregulated in PC. Next, we used stable isotope dilution multiple-reaction monitoring mass spectrometry (SID- MRM-MS) for targeted quantitation of these 21 metabolite markers in plasma samples obtained from patients that were diagnosed with PC and compared the profiles to normal controls with a similar median age and near uniform gender distribution. The PC patient samples (*N* = 59) were made available through the Indivumed repository at the MedStar-Georgetown University hospital while the normal controls (*N* = 48) were recruited at University of Rochester under approved IRB protocols. We also included plasma samples obtained from patients diagnosed with Type 2 diabetes (*N* = 19) or colorectal cancer (*N* = 66) as control cohorts to evaluate specificity of the biomarker panel.

The ten metabolite biomarkers delineated in this study using literature curation were found to be significantly dysregulated in PC patients as compared to normal controls. However, five of the metabolites (Lyso PC18:2, lactate, choline, lysine and asparagine) were not concordant across reported studies (Tables 1 and 2). Three metabolites, alanine [63, 64], threonine [63, 65], and tyrosine [63, 65], were found to be concordant with results from our study as well as across reported literature. On the other hand, 3-hydroxybuyterate [38, 64] and palmitate [57, 66], were reported to be down-regulated in PC across reported studies; however these metabolites were significantly up-regulated in PC patient samples upon analysis in our laboratory, with fold change values of 6.9 and 2.3 fold, respectively. These findings therefore, would need further investigations and cross-validation. Additionally, conflicting results in published literature further emphasize the need for a centralized biomarker repository to facilitate cross-laboratory and cross-platform evaluation of classifiers. One of the limitations of the meta-analysis could stem from the fact that PC and CRC samples used in our study represented localized and early stage disease while the data from the surveyed literature search included all stages, potentially lending to analytical variability.

In this study we used, CRC (as a cancer disease control) and T2DM (as a non-cancer control) cohorts for testing biomarker specificity. Several metabolite markers (eight of ten) overlapped between pancreatic cancer and colorectal cancer including, LysoPC (18:2), alanine, choline, tyrosine, lysine, and 3-hydroxybutyrate suggesting a generic metabolic perturbation pattern that underscores a cancerous metabotype. Furthermore, all of these metabolites have the same direction of regulation in the two cancer groups although their fold changes vary when compared to the normal controls.

Although T2DM has been intricately linked to the onset of PC [48], the degree of overlap between the metabolic markers between the two disease was much less (around 10–15%). The T2DM cohort used in this study was much younger and obtained from an Asian population as compared to the NCs and the PC cases which were primarily Caucasian; this is a potential weakness of the study cohort although we performed statistical analysis to rule out bias in resultant AUCs for different comparative groups. Additionally, a review of literature reporting metabolite profiling of NC vs T2DM using diverse populations show partial overlap with the markers delineated in this study [67–70]. These results emphasize the need to include related disease control cohorts that share commonalities with oncogenic progression of pancreatic cancer; these would warrant inclusion of other cancer cohorts as cancer disease controls but also those deemed to be high risk cohorts of PC such as patients diagnosed with chronic pancreatitis or with non-malignant precursor lesions of the pancreas.

Several signaling pathways that are altered during carcinogenesis are known to impact metabolic processes [71]. Given that several genetic mutations and signaling pathways overlap between different malignancies, it is reasonable to assume that this would result in shared metabolic dysregulations. Therefore, inclusion of cancer disease control and high-risk cohorts for PC biomarker studies is critical for eliminating non-specific metabolites that would otherwise diminish the clinical utility of the biomarker panel for PC diagnosis.

**Figure 8:**
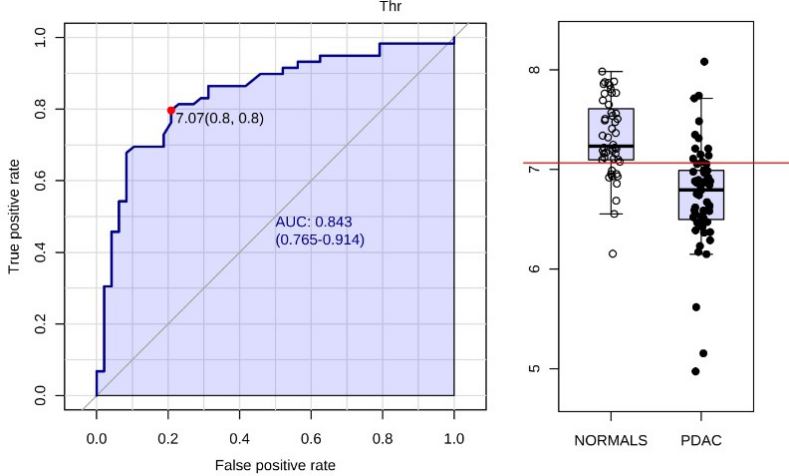
Receiver-operating characteristic (ROC) curve and for PC (*n* = 59) vs NC (*n* = 48) metabolite threonine yields AUC = 0.843

While we used CRC and T2DM as control for specificity, some papers from our literature survey included chronic pancreatitis (CP) or benign pancreatic conditions in their study. Overlapping biomarkers between chronic pancreatitis and our literature derived panel included lactate, lysine, histidine, glutamine, glutamate, and alanine, which were found elevated in CP vs NC [64]. Additionally, a comparison of CP and PC diagnostic groups yielded 3-hydroxybutyrate, lactate, valine, tyrosine, phenylalanine, lysine, isoleucine, histidine, glutamine, glutamate, and alanine as being up-regulated in chronic pancreatitis [64]. Comparison of PC and benign pancreatitis cases showed upregulation of 3-hydroxybutyrate and phenylalanine in PC, while lysine, asparagine, and threonine were down-regulated [44]. Threonine emerged as a specific marker for PC, although we could not include 1,5-anhydro-d-gluticol, CA19-9, and PC-594 in this analysis. The AUC for NC vs PC for threonine was 0.84 thus emphasizing the need for continued efforts to delineate a biomarker panel with high specificity for PC (Figure 8).

Discovery and validation of novel and robust biomarkers is integral to advance personalized medicine initiative. However, there is huge gap between reported cancer biomarker studies and their translation into potential clinical use [72]. Advancing the translation of biomarkers from a scientific discovery to regulatory approvals for clinical implementation would require a concerted effort [73]. This includes a) improvements in guidelines for designing biomarker development studies for evaluation of biomarker specificity and sensitivity; b) availability of high quality bio-specimens (with clinical annotations) by controlling sample collection and storage procedures; c) application of scientific, analytical and statistical rigor to the study design; d) standardization of data reporting guidelines for biomarker discovery studies thus enabling meta-analyses; e) data sharing to facilitate cross-platform an inter-laboratory reproducibility studies; f) external biomarker validation studies performed with GLP compliance [74]. Collectively, these measures would maximize the development of robust biomarker panels that would be ready for clinical testing; in addition this would also augment testing a combination of “omics” markers that would form better classifiers. It would also lead to mechanistic studies aimed at discerning how pathway perturbations lead to the observed disease phenotype. Understanding of biochemical alterations that underscore cancer progression, reflected as biomarkers, allows for a more practical way of identifying molecular targets that can be used for customized therapeutic development.

## MATERIALS AND METHODS

### Databases used

**Figure 9:**
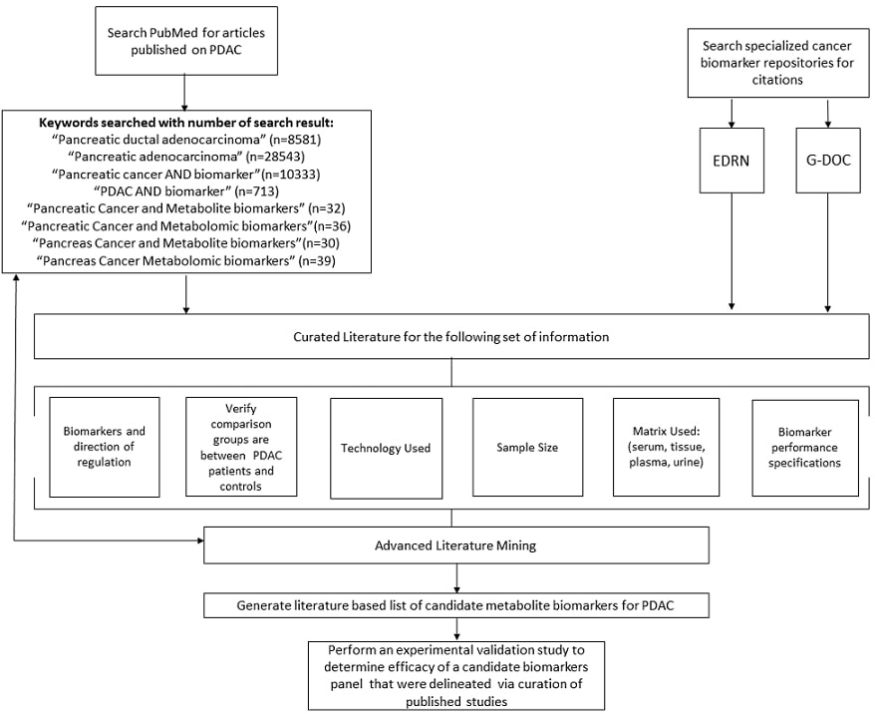
Schema for curating metabolites mined from literature search and meta- analyses

A large number of studies related to pancreatic cancer biomarkers have been indexed on multiple platforms. In order to identify relevant metabolomics studies, we focused our literature searches to PubMed, as well as the biomarker data repositories EDRN (Early Detection Research Network) and G-DOC (Georgetown Database of Cancer). PubMed is a database that contains abstract and full-text citations for biomedical literature from MEDLINE, life science journals, and online books, and is maintained by United States National Library of Medicine (NLM) at the National Institute of Health (NIH). EDRN, led by the National Cancer Institute (NCI), is a collaboration focused on the discovery and clinical application of cancer biomarkers. G-DOC is a platform that combines data integration and integrative knowledge discovery for oncological and translational research communities. In our approach (Figure 9), “pancreatic cancer” is searched in PubMed, which generates over 80,000 results. To narrow down the search results, different permutations of keywords pertaining to PC biomarkers were searched. As of December 2016 the eight keywords that returned results for metabolomics based biomarker studies of PC that have been cited in this study are listed, along with number of search results generated (‘Keywords Used’, Figure 9). On the other hand, “pancreatic ductal adenocarcinoma” and “pancreatic cancer” were used as search terms in the two previously discussed cancer biomarker repositories, G-DOC and EDRN.

Not all of the eight keywords used for searching PubMed generate results that are relevant for this study and hence were parsed. For instance, some studies focus on the structural biology of a metabolite while other studies had a bioinformatics focus. Each PubMed paper was manually curated for parsing information and determining suitability for the proposed meta-analysis.

### Manual curation procedures and criteria for including metabolites

The second screening step involves mining literature for the name of the metabolite and verifying contextual data for which it was discovered in previous studies. Each metabolite recorded in the final literature search must be strictly curated for the following criteria. Firstly, only studies with human-based matrices (urine, cell lines, tissue, plasma, serum) are allowed; no animal studies or animal to human comparative studies were included. Metabolites derived from the comparing of pancreatic cancer patients vs. normal patients, or controls were segregated from other comparisons. The sample size, technology used in biomarker discovery (i.e. mass spectrometry, NMR, etc.) and most importantly, and the direction of regulation of the biomarker were documented. We observed that not all studies found had information pertaining to all these categories emphasizing the need to standardize reporting guidelines for biomarker studies. An additional author-specific search was accomplished by mining the bibliography of different studies for additional relevant papers that may not have appeared in the initial PubMed search. Review articles found on PubMed were also cross-referenced, in order to verify that metabolite data were recorded correctly.

### Study participant and samples

A total of 192 plasma samples were analyzed with four diagnostic groups: PC, CRC, T2DM, and normal controls. PC (*n* = 59) and CRC (*n* = 66) samples were made available through the Indivumed repository at the MedStar-Georgetown University hospital protocols. PC and CRC patients were candidates for curative surgery and hence represented previously untreated early stages of cancer. Normal controls (*n* = 48) were recruited as a part of the Rochester aging study (RAS) under approved IRB protocols [75]. Plasma samples from patients with a diagnosis of T2DM (*n* = 19) (without any co-morbidity) were collected under approved IRB protocols in Qatar University [76, 77]. Clinical and demographic characteristics of the cohort as age, sex, ethnicity were recorded (Table 3). All samples were collected under 12 hour fasting conditions using stringent procedures for collection and storage thus minimizing confounding by pre-analytical variables on downstream MS analyses. Other factors and coexisting conditions such as BMI, jaundice, diabetes and smoking, alcohol traits for each subject were also annotated.

**Table 3:** Demographic details of the study participants

	PC (*n* = 59)	CRC (*n* = 66)	T2DM (*n* = 19)	Normal (*n* = 48)
Median Age	74.2	63.45	55	79
Ethnicity				
Caucasian	33	46	0	47
African American	13	11	3	1
Asian	7	8	16	0
Hispanic	2	0	0	0
Other	4	1	0	0
Gender				
Male	28	31	17	25
Female	31	35	2	23
Type II Diabetes	22	6	19	5
Mean BMI	25.072	25.829	29.525	26.175
Alcohol	20	33	0	35
Smoking	29	37	12	4
Jaundice	27	0	0	0

### Targeted liquid chromatography-mass spectrometry

Targeted analyses were performed using plasma samples on a Acquity UPLC (Waters Corporation, USA) online with a triple quadrupole MS (Xevo TQ-S Waters Corporation, USA) operating in the MRM mode. Different classes of metabolites such as amino acids, biogenic amines, glycerophospholipids, acylcarnitines, sphingolipids and sugars were analyzed using AbsoluteIDQ^®^ p180 kit (Biocrates Life Sciences AG, Innsbruck, Austria) as detailed by the manufacturer as described previously [75].

We also performed targeted mass spectrometry based quantification for 3-hydroxybutyrate, choline, lactate, palmitate, using a separate extraction. 15 ul of serum/plasma were mixed with 95 ul methanol containing 500 nM each of 17 isotopically labeled internal standards (Cambridge Isotope Labs product number MSK-A2-1.2) and vortexed for 20 seconds. After adding 190 ul dichloromethane, samples were again vortexed for 20 seconds. Finally, 60 ul water were added and the samples were vortexed for 10 min. at 4C, then centrifuged at 8,000 × g for 10 min. at 4°C. The two solvent layers were collected separately and dried under vacuum, then stored at −80C. The top (polar) layer was resuspended in 100 ul water and 1 ul was injected for LC/MS analysis as described [78]. The bottom (lipid) layer was resuspended in 50 ul 65:30:5 acetonitrile/isopropanol/water (v/v/v) and 5 ul was injected for LC/MS analysis as described [79].

### Statistical and bioinformatics analysis

Statistical analysis of the MS data were performed using MetaboAnalyst 3.0 [80]. Initially we used the statistical analysis module of MetaboAnalyst 3.0. Data were log transformed before performing t-statistics to delineate significant metabolites using fold change and test of significance as thresholds. Metabolites with an adjusted *p*-value of less than 0.05 and a fold change either less than or equal to 0.7 or greater than 1.8 were deemed significant for constructing a binary classifier using ROC analysis module of MetaboAnalyst v 3.0. Box plots were plotted using in-house R scripts. Plasma index metabolite index was calculated using a multinomial logistic regression model in R as described previously [81].

## SUPPLEMENTARY MATERIALS FIGURES AND TABLES







## Abbreviations

PC: (Pancreatic Cancer)
NC: (Normal Control)
CRC: (Colorectal Cancer)
T2DM: (Type II Diabetes)
CP: (Chronic Pancreatitis).
