# Supplementary material for: Metabolomic biomarkers of pancreatic cancer: a meta-analysis study
Source: Oncotarget. 2017 Aug 18;8(40):68899–915. doi: 10.18632/oncotarget.20324 (PMC5620306; doi:10.18632/oncotarget.20324)
Supplement: Supplementary file 1 [file oncotarget-08-68899-s001.pdf]

Metabolomic biomarkers of pancreatic cancer: a meta-analysis study

SUPPLEMENTARY MATERIALS

Supplementary Table 1: Blood based (serum or plasma) metabolites with no overlap across independent biomarker studies of pancreatic cancer. See Supplementary_Table_1

Supplementary Table 2: Metabolite markers reported in different matrices (other than blood based). See Supplementary_Table_2
