# Supplementary material for: Metabolomic biomarkers of pancreatic cancer: a meta-analysis study
Source: Oncotarget. 2017 Aug 18;8(40):68899–915. doi: 10.18632/oncotarget.20324 (PMC5620306; doi:10.18632/oncotarget.20324)
Supplement: Supplementary file 2 [file oncotarget-08-68899-s002.docx]

Supplementary table 1: Blood based (serum or plasma) metabolites with no overlap across independent biomarker studies of pancreatic cancer.

| **Biomarker** | **Comparison Groups** | **Instrument** | **Matrix** | **Reference (PMID)** |
| --- | --- | --- | --- | --- |
| 3-hydroxybutyrate ↑ | Pancreatic cancer (n=43) vs Benign pancreatic conditions (n=41) | H NMR | serum | 21098649 |
| 3-hydroxyisovalerate ↓ | Pancreatic cancer (n=17) vs Healthy (n=23) | 1H NMR | Serum | 21505807 |
| 3-methyl-2-oxovalerate ↑ | Pancreatic cancer (n=43) vs Benign pancreatic conditions (n=41) | H NMR | serum | 21098649 |
| 4-Pyridoxate ↓ | Pancreatic cancer (n=43) vs Benign pancreatic conditions (n=41) | H NMR | serum | 21098649 |
| Acetone ↑ | Pancreatic cancer (n=19) vs Healthy (n=20) vs Chronic pancreatitis (n=20) | H-NMR | Blood/plasma | 22613268 |
|  | Pancreatic cancer (n=43) vs Benign pancreatic conditions (n=41) | H NMR | serum | 21098649 |
| aconitic acid ↑ | Pancreatic cancer (n=20) vs Healthy (n=9) | GC/MS | serum | DOI: 10.1007/s11306-010-0224-9 |
| Arachidonic acid ↑ | Pancreatic cancer (n=5) vs Healthy (n=2) vs Chronic pancreatitis (n=3) | GC-TOF-MS | plasma | 20143319 |
| Argininea | Pancreatic cancer (n=360) vs Healthy (n=8372) vs Chronic pancreatitis (n=28) | HPLC-ESI-MS | plasma | 26133769 |
|  | Pancreatic cancer (n=43) vs Benign pancreatic conditions (n=41) | H NMR | serum | 21098649 |
| Asparaginea | Pancreatic cancer (n=43) vs Benign pancreatic conditions (n=41) | H NMR | serum | 21098649 |
| betaine ↓ | Pancreatic cancer (n=200) vs Healthy (n=200) | LC–TOFMS and GC–TOFMS | plasma | 25429707 |
| CA19-9 ↑ | Pancreatic cancer (105) vs Non-pancreatic malignancies (n=70) vs Benign pancreatic diseases (n=30) | electrochemiluminescence immunoassay on the Roche Elecsys 1010/2010 and MODULAR ANALYTICS E170 | serum | 18384788 |
| CEA ↑ | Pancreatic cancer (105) vs Non-pancreatic malignancies (n=70) vs Benign pancreatic diseases (n=30) | electrochemiluminescence immunoassay on the Roche Elecsys 1010/2010 and MODULAR ANALYTICS E170 | serum | 18384788 |
| Cholesterol ↑ | Pancreatic cancer (n=5) vs Healthy (n=2) vs Chronic pancreatitis (n=3) | GC-TOF-MS | plasma | 20143319 |
| Cholylglycine ↑ | Pancreatic cancer (n=5) vs Healthy (n=2) vs Chronic pancreatitis (n=3) | HILIC-LC/MS RP-LC/MS | plasma | 20143319 |
| citrate ↓ | Pancreatic cancer (n=19) vs Healthy (n=20) vs Chronic pancreatitis (n=20) | H-NMR | Blood/plasma | 22613268 |
| citrulline ↓ | Pancreatic cancer (n=360) vs Healthy (n=8372) vs Chronic pancreatitis (n=28) | HPLC-ESI-MS | plasma | 26133769 |
| creatine ↓ | Pancreatic cancer (n=43) vs Benign pancreatic conditions (n=41) | H NMR | serum | 21098649 |
| creatinine ↑ | Pancreatic cancer (n=17) vs Healthy (n=23) | 1H NMR | Serum | 21505807 |
| CS242 ↑ | Pancreatic cancer (105) vs Non-pancreatic malignancies (n=70) vs Benign pancreatic diseases (n=30) | electrochemiluminescence immunoassay on the Roche Elecsys 1010/2010 and MODULAR ANALYTICS E170 | serum | 18384788 |
| decanoic acid ↓ | Pancreatic cancer (n=20) vs Healthy (n=9) | GC/MS | serum | DOI: 10.1007/s11306-010-0224-9 |
| Deoxycholylglycine ↑ | Pancreatic cancer (n=5) vs Healthy (n=2) vs Chronic pancreatitis (n=3) | HILIC-LC/MS RP-LC/MS | plasma | 20143319 |
| Dimethylamine (DMA) ↑ | Pancreatic cancer (n=19) vs Healthy (n=20) vs Chronic pancreatitis (n=20) | H-NMR | Blood/plasma | 22613268 |
|  | Pancreatic cancer (n=43) vs Benign pancreatic conditions (n=41) | H NMR | serum | 21098649 |
| D-sphingosine ↓ | Pancreatic cancer (n=40) vs Healthy (n=40) | LC-MS/MS | serum | 26735340 |
| Erythritol ↑ | Pancreatic cancer (n=5) vs Healthy (n=2) vs Chronic pancreatitis (n=3) | GC-TOF-MS | plasma | 20143319 |
| ethanol ↓ | Pancreatic cancer (n=43) vs Benign pancreatic conditions (n=41) | H NMR | serum | 21098649 |
| formate ↑ | Pancreatic cancer (n=43) vs Benign pancreatic conditions (n=41) | H NMR | serum | 21098649 |
| glucose ↑ | Pancreatic cancer (n=43) vs Benign pancreatic conditions (n=41) | H NMR | serum | 21098649 |
| glucose plus triglycerides ↑ | Pancreatic cancer (n=14) vs Healthy (n=14) | 1H NMR, TOCSY, HMQC or HSQC | serum | 22422139 |
| Glutamate ↑ | Pancreatic cancer (n=43) vs Benign pancreatic conditions (n=41) | H NMR | serum | 21098649 |
| Glutamine ↓ | Pancreatic cancer (n=43) vs Benign pancreatic conditions (n=41) | H NMR | serum | 21098649 |
| glyceric acid ↓ | Pancreatic cancer (n=20) vs Healthy (n=9) | GC/MS | serum | DOI: 10.1007/s11306-010-0224-9 |
| glycerol ↓ | Pancreatic cancer (n=43) vs Benign pancreatic conditions (n=41) | H NMR | serum | 21098649 |
| high density lipoprotein (HDL) ↓ | Pancreatic cancer (n=19) vs Healthy (n=20) vs Chronic pancreatitis (n=20) | H-NMR | Blood/plasma | 22613268 |
| homogentistic acid ↑ | Pancreatic cancer (n=20) vs Healthy (n=9) | GC/MS | serum | DOI: 10.1007/s11306-010-0224-9 |
| Hydrocinnamic acid ↓ | Pancreatic cancer (n=5) vs Healthy (n=2) vs Chronic pancreatitis (n=3) | HILIC-LC/MS RP-LC/MS | plasma | 20143319 |
| hydroxyoctanoic acid ↑ | Pancreatic cancer (n=20) vs Healthy (n=9) | GC/MS | serum | DOI: 10.1007/s11306-010-0224-9 |
| Inosine ↓ | Pancreatic cancer (n=5) vs Healthy (n=2) vs Chronic pancreatitis (n=3) | HILIC-LC/MS RP-LC/MS | plasma | 20143319 |
| lauric acid ↓ | Pancreatic cancer (n=20) vs Healthy (n=9) | GC/MS | serum | DOI: 10.1007/s11306-010-0224-9 |
| LDL ↓ | Pancreatic cancer (n=19) vs Healthy (n=20) vs Chronic pancreatitis (n=20) | H-NMR | Blood/plasma | 22613268 |
| L-glycine ↓ | Pancreatic cancer (n=20) vs Healthy (n=9) | GC/MS | serum | DOI: 10.1007/s11306-010-0224-9 |
| Lysine ↓ | Pancreatic cancer (n=43) vs Benign pancreatic conditions (n=41) | H NMR | serum | 21098649 |
| LysoPC(16:0) ↑ | Pancreatic cancer (n=5) vs Healthy (n=2) vs Chronic pancreatitis (n=3) | HILIC-LC/MS | plasma | 20143319 |
| Mannose ↑ | Pancreatic cancer (n=43) vs Benign pancreatic conditions (n=41) | H NMR | serum | 21098649 |
| margaric acid ↓ | Pancreatic cancer (n=20) vs Healthy (n=9) | GC/MS | serum | DOI: 10.1007/s11306-010-0224-9 |
| Methanol ↓ | Pancreatic cancer (n=43) vs Benign pancreatic conditions (n=41) | H NMR | serum | 21098649 |
| methylguanidine ↑ | Pancreatic cancer (n=200) vs Healthy (n=200) | LC–TOFMS and GC–TOFMS | plasma | 25429707 |
| MIC-1 ↑ | Pancreatic cancer (n=50) vs Healthy (n=50) vs Chronic pancreatitis (n=50) | ELISA | serum | 16428484 |
| myristic acid ↓ | Pancreatic cancer (n=20) vs Healthy (n=9) | GC/MS | serum | DOI: 10.1007/s11306-010-0224-9 |
| N-acetyl glycoprotein (NAG) ↑ | Pancreatic cancer (n=19) vs Healthy (n=20) vs Chronic pancreatitis (n=20) | H-NMR | Blood/plasma | 22613268 |
| n-acetyltyrosine ↑ | Pancreatic cancer (n=20) vs Healthy (n=9) | GC/MS | serum | DOI: 10.1007/s11306-010-0224-9 |
| n-caprylic acid ↓ | Pancreatic cancer (n=43) vs Healthy (n=42) | GC/MS | Serum | 23542803 |
| N-Methylalanine ↑ | Pancreatic cancer (n=5) vs Healthy (n=2) vs Chronic pancreatitis (n=3) | GC-TOF-MS | plasma | 20143319 |
| nonanoic acid ↓ | Pancreatic cancer (n=43) vs Healthy (n=42) | GC/MS | Serum | 23542803 |
| octanoic acid ↓ | Pancreatic cancer (n=20) vs Healthy (n=9) | GC/MS | serum | DOI: 10.1007/s11306-010-0224-9 |
| oleanolic acid ↑ | Pancreatic cancer (n=40) vs Healthy (n=40) | LC-MS/MS | serum | 26735340 |
| paraxanthine ↓ | Pancreatic cancer with cachexia (n=9) vs Pancreatic cancer without cachexia (n=12) | GC/MS | blood | 25411961 |
| PC (34:2) ↑ | Pancreatic cancer (n=5) vs Healthy (n=2) vs Chronic pancreatitis (n=3) | HILIC-LC/MS | plasma | 20143319 |
| PE(26:0) ↑ | Pancreatic cancer (n=5) vs Healthy (n=2) vs Chronic pancreatitis (n=3) | RP-LC/MS | plasma | 20143319 |
| Phenylalanine ↑ | Pancreatic cancer (n=43) vs Benign pancreatic conditions (n=41) | H NMR | serum | 21098649 |
| Proline ↓ | Pancreatic cancer (n=360) vs Healthy (n=8372) vs Chronic pancreatitis (n=28) | HPLC-ESI-MS | plasma | 26133769 |
|  | Pancreatic cancer (n=43) vs Benign pancreatic conditions (n=41) | H NMR | serum | 21098649 |
| PtdCho 18:0/18:2 ↓ | Pancreatic cancer (n=40) vs Healthy (n=50) | FI-FTICR-MS | serum | 24024929 |
| serine ↑ | Pancreatic cancer (n=360) vs Healthy (n=8372) vs Chronic pancreatitis (n=28) | HPLC-ESI-MS | plasma | 26133769 |
| sphingomyelin d18:1/24:0) ↓ | Pancreatic cancer (n=40) vs Healthy (n=50) | FI-FTICR-MS | serum | 24024929 |
| stearic acid ↓ | Pancreatic cancer (n=20) vs Healthy (n=9) | GC/MS | serum | DOI: 10.1007/s11306-010-0224-9 |
| taurine ↑ | Pancreatic cancer (n=14) vs Healthy (n=14) | 1H NMR, TOCSY, HMQC or HSQC | serum | 22422139 |
| taurochenodeoxycholate ↑ | Pancreatic cancer (n=40) vs Healthy (n=40) | LC-MS/MS | serum | 26735340 |
| Taurocholic acid ↑ | Pancreatic cancer (n=5) vs Healthy (n=2) vs Chronic pancreatitis (n=3) | HILIC-LC/MS RP-LC/MS | plasma | 20143319 |
| Tauroursodeoxycholic acid ↑ | Pancreatic cancer (n=5) vs Healthy (n=2) vs Chronic pancreatitis (n=3) | RP-LC/MS | plasma | 20143319 |
| thiodiglycolic acid ↑ | Pancreatic cancer (n=20) vs Healthy (n=9) | GC/MS | serum | DOI: 10.1007/s11306-010-0224-9 |
| Threonine ↓ | Pancreatic cancer (n=43) vs Benign pancreatic conditions (n=41) | H NMR | serum | 21098649 |
| triglyceride ↑ | Pancreatic cancer (n=17) vs Healthy (n=23) | 1H NMR | Serum | 21505807 |
| trimethylamine- N-oxide ↓ | Pancreatic cancer (n=17) vs Healthy (n=23) | 1H NMR | Serum | 21505807 |
| Tryptamine ↓ | Pancreatic cancer (n=5) vs Healthy (n=2) vs Chronic pancreatitis (n=3) | RP-LC/MS | plasma | 20143319 |
| Tryptophan ↓ | Pancreatic cancer (n=360) vs Healthy (n=8372) vs Chronic pancreatitis (n=28) | HPLC-ESI-MS | plasma | 26133769 |
|  | Pancreatic cancer (n=43) vs Benign pancreatic conditions (n=41) | H NMR | serum | 21098649 |
| uric acid ↓ | Pancreatic cancer (n=20) vs Healthy (n=9) | GC/MS | serum | DOI: 10.1007/s11306-010-0224-9 |
| Urea ↓ | Pancreatic cancer (n=43) vs Benign pancreatic conditions (n=41) | H NMR | serum | 21098649 |
|  | Pancreatic cancer (n=20) vs Healthy (n=9) | GC/MS | serum | DOI: 10.1007/s11306-010-0224-9 |
| very low density lipoprotein (VLDL) ↑ | Pancreatic cancer (n=19) vs Healthy (n=20) vs Chronic pancreatitis (n=20) | H-NMR | Blood/plasma | 22613268 |
| ^a^Metabolites that were found in multiple articles but with different regulations | | | | |
