# Supplementary material for: Metabolomic biomarkers of pancreatic cancer: a meta-analysis study
Source: Oncotarget. 2017 Aug 18;8(40):68899–915. doi: 10.18632/oncotarget.20324 (PMC5620306; doi:10.18632/oncotarget.20324)
Supplement: Supplementary file 3 [file oncotarget-08-68899-s003.docx]

Supplementary table 2: Metabolite markers reported in different matrices (other than blood based).

| **Biomarker** | **Comparison Groups** | **Instrument** | **Matrix** | **Reference (PMID)** |
| --- | --- | --- | --- | --- |
| 1-Methylnicotinamide (↑) | Pancreatic cancer patients (n = 32) vs benign pancreatic conditions (n=25) | H-NMR | urine | 23096698 |
| 2-amino-3-methylimidazo[4,5-f]quinoxaline-8-carboxylic acid (↑) | Pancreatic cancer patients (n = 4) vs normal controls (n=4) | HPLC | urine | 26918625 |
| 2-Hydroxyisobutyrate (↑) | Pancreatic cancer patients (n = 32) vs benign pancreatic conditions (n=25) | H-NMR | urine | 23096698 |
| 2-phenylacetamide (↑) | Pancreatic cancer patients (n=33) vs normal controls (n=54) | 1H NMR | urine | 22066465 |
| 3-hydroxyisovalerate (↓) | Pancreatic cancer patients (n=33) vs normal controls (n=54) | 1H NMR | urine | 22066465 |
| 4-Hydroxyphenylacetate (↑) | Pancreatic cancer patients (n = 32) vs benign pancreatic conditions (n=25) | H-NMR | urine | 23096698 |
| 4-Pyridoxate (↑) | Pancreatic cancer patients (n = 32) vs benign pancreatic conditions (n=25) | H-NMR | urine | 23096698 |
| Acetate (↑) | PDAC cell lines (n=8) vs immortalized pancreatic cell line (n=1) | H MRS, MRSI | tissue | 25370468 |
| Acetoacetate (↑) | Pancreatic cancer patients (n=33) vs normal controls (n=54) | 1H NMR | urine | 22066465 |
| Acetone (↑) | Pancreatic cancer patients (n = 32) vs benign pancreatic conditions (n=25) | H-NMR | urine | 23096698 |
| Aminobutyrate (↑) | Pancreatic cancer patients (n = 32) vs benign pancreatic conditions (n=25) | H-NMR | urine | 23096698 |
| Aspartate (↑) | non-PDAC cell lines (n=200) vs. PDAC cell lines in glycolytic and lipogenic phases (n=38) | CE-TOF-MS | saliva | 20300169 |
| Choline (↑) | Pancreatic cancer patients (n = 32) vs benign pancreatic conditions (n=25) | H-NMR | urine | 23096698 |
| cis-Aconitate (↑) | Pancreatic cancer patients (n = 32) vs benign pancreatic conditions (n=25) | H-NMR | urine | 23096698 |
| Citrate (↓) | Pancreatic cancer patients (n=33) vs normal controls (n=54) | 1H NMR | urine | 22066465 |
| Creatine (↑) | PDAC cell lines (n=8) vs immortalized pancreatic cell line (n=1) | H MRS, MRSI | tissue | 25370468 |
| Creatinine (↓) | Pancreatic cancer patients (n=33) vs normal controls (n=54) | 1H NMR | urine | 22066465 |
| Dimethylamine (↑) | Pancreatic cancer patients (n = 32) vs benign pancreatic conditions (n=25) | H-NMR | urine | 23096698 |
| Flavine Adenine Dinucleotide (↓) | Pancreatic cancer cell lines (n = 190) vs non-PDAC cell lines (n=200) | IP-UPLC and MS/MS | tissue | 26216984 |
| Fucose (↑) | Pancreatic cancer patients (n = 32) vs benign pancreatic conditions (n=25) | H-NMR | urine | 23096698 |
| Glucose (↑) | Pancreatic cancer patients (n=33) vs normal controls (n=54) | 1H NMR | urine | 22066465 |
| Glucose (↑) | Pancreatic cancer patients (n = 32) vs benign pancreatic conditions (n=25) | H-NMR | urine | 23096698 |
| Glutamate (↑) | non-PDAC cell lines (n=200) vs. PDAC cell lines in glycolytic and lipogenic phases (n=38) | CE-TOF-MS | saliva | 20300169 |
| Glutamate (↑) | PDAC cell lines (n=8) vs immortalized pancreatic cell line (n=1) | H MRS, MRSI | tissue | 25370468 |
| Glutamine (↑) | non-PDAC cell lines (n=200) vs. PDAC cell lines in glycolytic and lipogenic phases (n=38) | CE-TOF-MS | saliva | 20300169 |
| Glyceraldehyde-3-phosphate (↑) | Pancreatic cancer cell lines (n = 190) vs non-PDAC cell lines (n=200) | IP-UPLC and MS/MS | tissue | 26216984 |
| Glycine (↓) | Pancreatic cancer patients (n=33) vs normal controls (n=54) | 1H NMR | urine | 22066465 |
| glycosphingolipid globotriaosylceramide (↑) | Pancreatic cancer patients (n = 27) vs normal controls (n=27) | Thin layer chromatography | tissue | 21788400 |
| GSH (↓) | Pancreatic cancer cell lines (n = 190) vs non-PDAC cell lines (n=200) | IP-UPLC and MS/MS | tissue | 26216984 |
| GSSG (↓) | Pancreatic cancer cell lines (n = 190) vs non-PDAC cell lines (n=200) | IP-UPLC and MS/MS | tissue | 26216984 |
| Hippurate (↓) | Pancreatic cancer patients (n=33) vs normal controls (n=54) | 1H NMR | urine | 22066465 |
| Hypoxanthine (↑) | Pancreatic cancer patients (n = 32) vs benign pancreatic conditions (n=25) | H-NMR | urine | 23096698 |
| isoleucine (↑) | non-PDAC cell lines (n=200) vs. PDAC cell lines in glycolytic and lipogenic phases (n=38) | CE-TOF-MS | saliva | 20300169 |
| Lactate (↑) | PDAC cell lines (n=8) vs immortalized pancreatic cell line (n=1) | H MRS, MRSI | tissue | 25370468 |
|  | Pancreatic cancer cell lines (n = 190) vs non-PDAC cell lines (n=200) | Ion-Pair (IP)-UPLC; Tandem MS (MS/MS) | tissue | 26216984 |
| Leucine (↑) | Pancreatic cancer patients (n=33) vs normal controls (n=54) | 1H NMR | urine | 22066465 |
|  | non-PDAC cell lines (n=200) vs. PDAC cell lines in glycolytic and lipogenic phases (n=38) | CE-TOF-MS | saliva | 20300169 |
| Linoleate (↑) | Pancreatic cancer patients (n=33) vs normal controls (n=54) | 1H NMR | urine | 22066465 |
| Margarate (↓) | Pancreatic cancer patients (n=33) vs normal controls (n=54) | 1H NMR | urine | 22066465 |
| Methanol (↓) | Pancreatic cancer patients (n = 32) vs benign pancreatic conditions (n=25) | H-NMR | urine | 23096698 |
| Myo-inositol (↑) | PDAC cell lines (n=8) vs immortalized pancreatic cell line (n=1) | H MRS, MRSI | tissue | 25370468 |
| Myristic Acid (↓) | Pancreatic cancer cell lines (n = 190) vs non-PDAC cell lines (n=200) | LC+GC-MS | tissue | 26216984 |
| NAD (↓) | Pancreatic cancer cell lines (n = 190) vs non-PDAC cell lines (n=200) | IP-UPLC and MS/MS | tissue | 26216984 |
| NADP (↓) | Pancreatic cancer cell lines (n = 190) vs non-PDAC cell lines (n=200) | IP-UPLC and MS/MS | tissue | 26216984 |
| NADPH (↓) | Pancreatic cancer cell lines (n = 190) vs non-PDAC cell lines (n=200) | IP-UPLC and MS/MS | tissue | 26216984 |
| O-acetylcarnitine (↑) | Pancreatic cancer patients (n = 32) vs benign pancreatic conditions (n=25) | H-NMR | urine | 23096698 |
| Oleate (↑) | Pancreatic cancer patients (n=33) vs normal controls (n=54) | 1H NMR | urine | 22066465 |
| Oleic Acid (↓) | Pancreatic cancer cell lines (n = 190) vs non-PDAC cell lines (n=200) | LC+GC-MS | tissue | 26216984 |
| Palmitate (↓) | Pancreatic tumor (n = 33) vs adjacent nontumor tissues (n=33) | UHPLC/MS/MS2, GC/MS | tissue | 23918603 |
| Palmitate (↓) | Pancreatic cancer patients (n=33) vs normal controls (n=54) | 1H NMR | urine | 22066465 |
| Palmlitoleic acid (↓) | Pancreatic cancer cell lines (n = 190) vs non-PDAC cell lines (n=200) | LC+GC-MS | tissue | 26216984 |
| PEP (↑) | Pancreatic cancer cell lines (n = 190) vs non-PDAC cell lines (n=200) | IP-UPLC and MS/MS | tissue | 26216984 |
| Phenylalanine (↑) | non-PDAC cell lines (n=200) vs. PDAC cell lines in glycolytic and lipogenic phases (n=38) | CE-TOF-MS | saliva | 20300169 |
| Pipecolic Acid (↑) | non-PDAC cell lines (n=200) vs. PDAC cell lines in glycolytic and lipogenic phases (n=38) | CE-TOF-MS | saliva | 20300169 |
| Serine (↑) | Pancreatic cancer cell lines (n = 190) vs non-PDAC cell lines (n=200) | IP-UPLC and MS/MS | tissue | 26216984 |
| Stearate (↓) | Pancreatic tumor (n = 33) vs adjacent nontumor tissues (n=33) | UHPLC/MS/MS2, GC/MS | tissue | 23918603 |
|  | Pancreatic cancer patients (n=33) vs normal controls (n=54) | 1H NMR | urine | 22066465 |
| Taurin (↑) | non-PDAC cell lines (n=200) vs. PDAC cell lines in glycolytic and lipogenic phases (n=38) | CE-TOF-MS | saliva | 20300169 |
|  | Pancreatic cancer patients (n = 32) vs benign pancreatic conditions (n=25) | H-NMR | urine | 23096698 |
| Threonine (↑) | Pancreatic cancer patients (n = 32) vs benign pancreatic conditions (n=25) | H-NMR | urine | 23096698 |
| trans-Aconitate (↑) | Pancreatic cancer patients (n = 32) vs benign pancreatic conditions (n=25) | H-NMR | urine | 23096698 |
| Trigonelline (↓) | Pancreatic cancer patients (n = 32) vs benign pancreatic conditions (n=25) | H-NMR | urine | 23096698 |
| Trimethylamine-N-oxide (↑) | Pancreatic cancer patients (n = 32) vs benign pancreatic conditions (n=25) | H-NMR | urine | 23096698 |
| Tryptophan (↑) | non-PDAC cell lines (n=200) vs. PDAC cell lines in glycolytic and lipogenic phases (n=38) | CE-TOF-MS | saliva | 20300169 |
| Tryptophan (↑) | Pancreatic cancer patients (n = 32) vs benign pancreatic conditions (n=25) | H-NMR | urine | 23096698 |
| valine (↑) | non-PDAC cell lines (n=200) vs. PDAC cell lines in glycolytic and lipogenic phases (n=38) | CE-TOF-MS | saliva | 20300169 |
| Xylose | Pancreatic cancer patients (n=33) vs normal controls (n=54) | 1H NMR | urine | 22066465 |
| Xylose (↑) | Pancreatic cancer patients (n = 32) vs benign pancreatic conditions (n=25) | H-NMR | urine | 23096698 |
